# Middle East respiratory syndrome coronavirus M protein suppresses type I interferon expression through the inhibition of TBK1-dependent phosphorylation of IRF3

**DOI:** 10.1038/emi.2016.33

**Published:** 2016-04-20

**Authors:** Pak-Yin Lui, Lok-Yin Roy Wong, Cheuk-Lai Fung, Kam-Leung Siu, Man-Lung Yeung, Kit-San Yuen, Chi-Ping Chan, Patrick Chiu-Yat Woo, Kwok-Yung Yuen, Dong-Yan Jin

**Affiliations:** 1 School of Biomedical Sciences, The University of Hong Kong, Pokfulam Hong Kong, China; 2 Department of Microbiology, The University of Hong Kong, Pokfulam, Hong Kong, China

**Keywords:** innate antiviral response, IRF3 activation, MERS coronavirus, RIG-I-like receptors, type I interferons

## Abstract

Middle East respiratory syndrome coronavirus (MERS-CoV) infection has claimed hundreds of lives and has become a global threat since its emergence in Saudi Arabia in 2012. The ability of MERS-CoV to evade the host innate antiviral response may contribute to its severe pathogenesis. Many MERS-CoV-encoded proteins were identified to have interferon (IFN)-antagonizing properties, which correlates well with the reduced IFN levels observed in infected patients and *ex vivo* models. In this study, we fully characterized the IFN-antagonizing property of the MERS-CoV M protein. Expression of MERS-CoV M protein suppressed type I IFN expression in response to Sendai virus infection or poly(I:C) induction. This suppressive effect was found to be specific for the activation of IFN regulatory factor 3 (IRF3) but not nuclear factor-κB. MERS-CoV M protein interacted with TRAF3 and disrupted TRAF3–TBK1 association leading to reduced IRF3 activation. M proteins from MERS-CoV and SARS-CoV have three highly similar conserved N-terminal transmembrane domains and a C-terminal region. Using chimeric and truncation mutants, the N-terminal transmembrane domains of the MERS-CoV M protein were found to be sufficient for its inhibitory effect on IFN expression, whereas the C-terminal domain was unable to induce this suppression. Collectively, our findings suggest a common and conserved mechanism through which highly pathogenic MERS-CoV and SARS-CoV harness their M proteins to suppress type I IFN expression at the level of TBK1-dependent phosphorylation and activation of IRF3 resulting in evasion of the host innate antiviral response.

## INTRODUCTION

Middle East respiratory syndrome coronavirus (MERS-CoV) was first identified in Saudi Arabia in September 2012 as a novel coronavirus that causes severe acute respiratory disease.^1^ Since then, this virus has caused recurrent outbreaks in the Arabian Peninsula and has spread, occasionally, to other parts of the world.^2, 3, 4, 5, 6, 7, 8, 9^ According to the World Health Organization, 1626 laboratory-confirmed cases were reported between September 2012 and 7 January 7 2016 with 586 related deaths in 26 countries.^10^ In particular, 186 people were infected and 36 were killed in one recent outbreak in South Korea.^10^ MERS-CoV is classified into lineage C of *Betacoronavirus* and is most phylogenetically related to two bat coronaviruses, HKU4 and HKU5, providing insight on its evolutionary origin.^11, 12^

MERS-CoV is a polycistronic positive-sense single-stranded RNA virus with a genome of ~30 Kb in size. The 5′ most two-thirds of MERS-CoV genome encodes polyproteins 1a and 1ab, which are further cleaved to yield 16 non-structural proteins, whereas the 3′ end of the genome encodes several structural or lineage-specific proteins.^13^ Upon infection, these proteins are expressed to facilitate viral replication and propagation in the host.^14^ MERS-CoV infection has been widely reported to mildly induce type I interferons (IFNs), including IFN-α and -β, in patients as well as in animal and cellular infection models.^15, 16, 17, 18, 19, 20, 21^ This has been attributed to the IFN-antagonizing property of some MERS-CoV-encoded proteins, which directly perturb the host IFN production mechanisms,^22, 23, 24, 25, 26^ lending support to the notion that MERS-CoV uses multiple strategies to evade the innate immune response.

In non-specialized epithelial cells as well as a subset of specialized immune cells that are susceptible to MERS-CoV infection,^16, 18, 27^ type I IFN production is an important part of the host innate immune response and is initiated by ubiquitously expressed cytoplasmic viral sensors in the retinoic acid-inducible gene-I (RIG-I)-like receptor (RLR) family in response to the detection of viral pathogen-associated molecular patterns such as double-stranded RNA (dsRNA).^28, 29^ Stimulated RLRs mobilize downstream signal transducers that lead to the activation of the transcription factors IFN regulatory factor 3 (IRF3) and nuclear factor-κB (NF-κB) that drive IFN-β expression.^28^ The transduction events within this signaling cascade are prone to negative regulation by many MERS-CoV proteins. In a comparative analysis of MERS-CoV structural and accessory proteins, it has been shown that M, ORF4a, ORF4b and ORF5 possess IFN-antagonizing properties.^22^ We, and others, have characterized the ORF4a protein as a dsRNA-binding protein that interferes with the activation of RLR by either a dsRNA ligand or the protein co-activator PACT.^24, 25^ However, the molecular mechanisms through which other MERS-CoV proteins manipulate the RLR signaling pathway to disrupt IFN-β expression have not been elucidated.

In this study, we focused on the characterization of the MERS-CoV M protein in IFN antagonism. Coronavirus M protein is a transmembrane glycoprotein localized predominantly to the Golgi complex and is required for virion assembly.^30, 31, 32^ MERS-CoV M protein is of particular interest because SARS-CoV M protein also inhibits IFN production through a mechanism by which the formation of TRAF3·TANK·TBK1/IKK-ɛ complex is impeded to ablate the activation of IRF3 transcription factor.^30^ In contrast, M protein encoded by human coronavirus HKU1 associated with common cold has no influence on IFN production.^32^ Here we reported that the MERS-CoV M protein also specifically inhibited IRF3 activation but not NF-κB signaling. MERS-CoV M protein was capable of interacting with TRAF3 adapter protein and hampered TRAF3–TBK1 interaction leading to diminished IRF3 activation. Using a chimeric protein containing the MERS-CoV M protein N-terminal transmembrane domains and a dormant SARS-CoV M protein C-terminal domain, we confirmed that the N-terminal transmembrane domains of MERS-CoV M protein sufficiently account for its inhibitory effect. Although another chimera containing SARS-CoV M protein N-terminal transmembrane domains and a MERS-CoV M protein C-terminal domain was fully competent in IFN antagonism, a truncation mutant lacking the functional first transmembrane domain of SARS-CoV M was not, suggesting that the C-terminal domain of the MERS-CoV M protein is largely dispensable for its immunosuppressive activity.

## MATERIALS AND METHODS

### Plasmids

The IFNβ-luc reporter plasmid and RIG-I expression plasmid are gifts from Professor Takashi Fujita (Kyoto University, Kyoto, Japan).^28^ The expression plasmids for TBK1, IRF3 and TRAF3 were generous gifts from Dr Genhong Cheng (University of California, Los Angeles, CA, USA),^33, 34^ whereas those for RIG-I N, IKK-ɛ and MAVS and IκB-α as well as IRF3-luc and κB-luc reporter plasmids have been described elsewhere.^30, 35, 36, 37^

Viral RNA was extracted from MERS-CoV-infected Vero-E6 cells. The M gene was PCR-amplified from complementary DNA and cloned into the *Eco*RI/*Xho*I sites of pCAGEN plasmid with the addition of a C-terminal V5-tag with the following primers: 5′-ATG TCT AAT ATG ACG CAA CTC ACT GA-3′ (forward) and 5′-AGC TCG AAG CAA TGC AAG TTC-3′ (reverse). The SARS-CoV M protein expression plasmid has been described elsewhere.^30, 32^ The expression plasmids for the SN and MN chimeras were constructed by assembly PCR with the following forward primers covering the breakpoints: 5′-AGG CTG TTT GCT CGT ACC CGC TCA TGG TGG TCA TTC AAT CCT GAG-3′ (SN) and 5′-CCG GCT GTT TAT GAG AAC TGG ATC AAT GTG GTC ATT CAA CCC A-3′ (MN). The reverse primers were complementary to their respective forward primers. The truncation mutant of the SN chimera lacking the first transmembrane domain was constructed using the forward primer: 5′-ATG GTA ACA CTT GCT TGT TTT GTG CT-3′.

### Antibodies

Mouse anti-FLAG (M2) and anti-β-actin antibodies were purchased from Sigma-Aldrich (St. Louis, MO, USA). Mouse anti-V5 and anti-HA (Y11) antibodies were purchased from Life Technologies (Grand Island, NY, USA) and Santa Cruz Biotechnology (Dallas, TX, USA), respectively. Rabbit anti-IRF3 and anti-phospho-IRF3 (Ser 386) antibodies were purchased from IBL-America (Minneapolis, MN, USA).

### Cell culture and Sendai virus

HEK-293 human embryonic kidney cells were maintained in Dulbecco's modified Eagle's medium with 10% fetal bovine serum (Life Technologies) at 37 °C in a humidified chamber supplemented with 5% carbon dioxide. Plasmid transfection was performed with GeneJuice (Merck Millipore; Billerica, MA, USA). poly(I:C) was purchased from Sigma-Aldrich and was transfected with Lipofectamine 2000 (Life Technologies). Sendai virus (Cantell strain) was purchased from American Type Culture Collection (Manassas, VA, USA).

### Reporter and protein assays

Dual-luciferase reporter assay, co-immunoprecipitation and western blotting were performed as previously described.^30, 38^ Particularly, relative luciferase activity in arbitrary units was calculated by normalizing firefly luciferase activity to *Renilla* luciferase activity recovered from cell lysate. Non-denaturing native polyacrylamide gel electrophoresis (PAGE) was performed as previously described.^36, 39, 40^

### Bioinformatic analysis

Sequence alignment was performed using Cluster Omega, an online tool based on the hidden Markov model,^41^ and hosted by the EMBL-EBI server (http://www.ebi.ac.uk/Tools/msa/clustalo/). Transmembrane domain prediction was performed using TMFinder, which considers hydrophobicity and helicity of the amino-acid sequence (http://tmfinder.research.sickkids.ca/).^42^

## RESULTS

### Inhibition of IFN expression by MERS-CoV M protein mediated through IRF3

**Figure 1:**
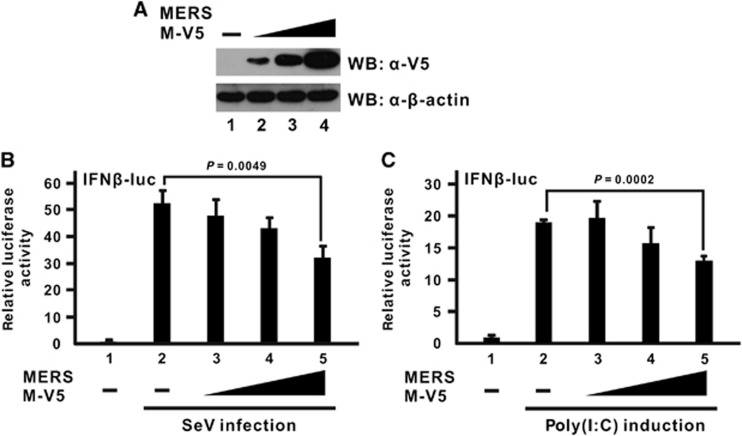
MERS-CoV M protein inhibits IFN-β expression stimulated by Sendai virus infection or poly(I:C) induction. Increasing doses of V5-tagged M protein expression plasmid (MERS M-V5) were transfected into HEK-293 cells. (**A**) Western blotting (WB) was performed with an anti-V5 antibody to confirm M protein expression in cell lysates. β-Actin was also used as an internal loading control. (**B**, **C**) M expression plasmid was co-transfected with a firefly luciferase reporter plasmid driven by the IFN-β promoter (IFNβ-luc) and a control *Renilla* luciferase reporter plasmid. After 24 h, cells were challenged with either Sendai virus infection (SeV; 100 hemagglutinating units per mL) in B or poly(I:C) induction (1 μL/mL) in C for 16 h before harvest for dual-luciferase reporter assay. Bars represent the mean of three biological replicates (*n*=3) and error bars indicate their s.d. The statistical significance between selected samples was evaluated using a two-tailed Student's *t*-test for unpaired samples with equal variance and *P*-value (*P*) was indicated.

To characterize the MERS-CoV M protein in terms of its IFN antagonism, M protein was ectopically expressed in cultured cells for functional assays (Figure 1A). A luciferase reporter construct driven by IFN-β promoter was used to reflect IFN-β promoter activity stimulated during infection. Sendai virus was used as a model virus to potently induce IFN expression in transfected cells. When increasing doses of M proteins were expressed in advance, a dose-dependent inhibition of IFN-β promoter activity was observed (Figure 1B; bars 3–5 compared with bar 2). A similar observation was also noted when synthetic dsRNA analog poly(I:C) was used as an alternative inducer that specifically stimulates the RLR pathway of IFN production (Figure 1C; bars 3–5 compared with bar 2). These two pieces of data are generally consistent with a previous report,^22^ and they further strengthen the current model of the IFN antagonism of MERS-CoV M protein.

**Figure 2:**
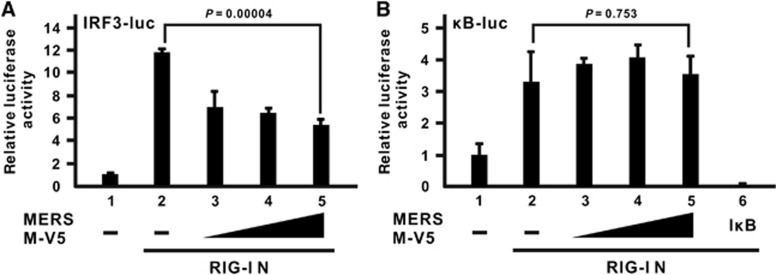
MERS-CoV M protein suppresses IRF3 activation but not NF-κB signaling. An expression plasmid for dominant-active RIG-I N mutant containing the N-terminal domain of RIG-I alone was used as a potent IFN inducer and co-transfected with an M expression plasmid and a firefly luciferase plasmid driven by tandem copies of (**A**) IRF3-binding elements (IRF3-luc) or (**B**) κB elements (κB-luc) as well as the control *Renilla* luciferase plasmid. A canonical NF-κB inhibitor IκB-α (IκB) was included as a positive control for the inhibition of NF-κB activity. Cells were collected at 40 h post transfection for dual-luciferase reporter assay. Bars represent the mean of three biological replicates (*n*=3) and error bars indicate their s.d. The statistical significance between selected samples was evaluated using a two-tailed Student's *t*-test for unpaired samples with equal variance and *P*-value (*P*) was indicated. retinoic acid-inducible gene-I, RIG-I.

Cellular IFN-β expression is under the control of multiple transcription factors, which work cooperatively to form a large enhanceosome complex.^43^ In particular, IRF3 and NF-κB are two transcription factors that are primarily activated by RLR signaling.^28, 44, 45^ MERS-CoV M protein has previously been shown to have no influence on NF-κB activation induced by Sendai virus infection.^22^ However, it remains to be seen whether the MERS-CoV M protein could preferentially inhibit IRF3 and NF-κB signaling after RIG-I activation. To address this issue, two different luciferase reporter constructs, in which tandem copies of either IRF3- or NF-κB-binding elements were inserted into their promoter region, were used. The truncation mutant of RIG-I known as RIG-I N that contains only the N-terminal CARD domain was chosen to be the inducer in these assays because it is constitutively active and highly competent to induce these two pathways.^28^ The MERS-CoV M protein was able to suppress the promoter activity of the IRF3-driven luciferase construct in a dose-dependent manner (Figure 2A; bars 3–5 compared with bar 2), but no inhibitory effect was observed with the NF-κB-driven construct (Figure 2B; bars 3–5 compared with bar 2) although the canonical inhibitor IκB-α could efficiently blunt its activation as a positive control (Figure 2B; bar 6 compared with bar 2). Hence, the suppressive effect of the MERS-CoV M protein is specific for IRF3 signaling but not NF-κB activation.

### Inhibition of IRF3 activation by the MERS-CoV M protein at TRAF3–TBK1 level

**Figure 3:**
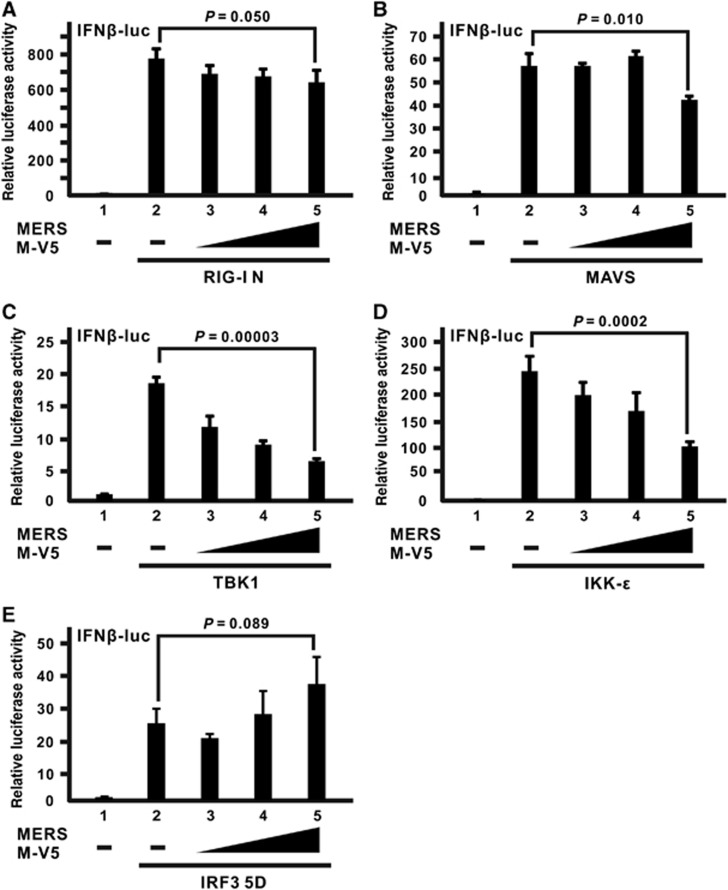
The inhibitory effect of the MERS-CoV M protein occurs upstream of IRF3 activation. (**A**–**E**) HEK-293 cells were transfected with increasing doses of the MERS M-V5 expression plasmid, a firefly luciferase reporter plasmid driven by IFN-β promoter (IFNβ-luc) and a control *Renilla* luciferase reporter plasmid, together with different expression plasmids of RLR pathway activators, RIG-I N in A, mitochondrial adapter MAVS in B, IRF3 kinases TBK1 and IKK-ɛ in C and D as well as a dominant-active IRF3 mutant (IRF3 5D) in E. Cells were collected after 40 h for dual-luciferase reporter assay. Bars represent the mean of three biological replicates (*n*=3) and error bars indicate their s.d. The statistical significance between selected samples was evaluated using a two-tailed Student's *t*-test for unpaired samples with equal variance and *P*-value (*P*) was indicated. Retinoic acid-inducible gene-I, RIG-I; RIG-I-like receptor, RLR.

To delineate the action point of the MERS-CoV M protein in IFN antagonism, we tested the ability of the M protein to inhibit the activation signal induced by different signal transducers of the RLR pathway individually. The activation signal will be mostly unaffected if the activation event mediated by that transducer is downstream of the action point where M protein exerts its inhibitory effect. As described above, RIG-I N is a constitutively active mutant that resembles immediate activation of virus sensor RIG-I after the detection of viral infection. Although the M protein could mildly suppress IFN-β promoter activation induced by RIG-I N at a marginally significant level, a dose-dependent effect was coarsely observed (Figure 3A; bars 3–5 compared with bar 2). A similar result was also obtained using MAVS as an activator (Figure 3B; bars 3–5 compared with bar 2), which is a mitochondrial adapter that diverts the activation signal from RIG-I to the IRF3 and NF-κB pathways.^44, 45, 46, 47^ When activators committed to the IRF3 pathway were used, greater inhibitory effects were observed, as in the cases of TBK1 (Figure 3C; bars 3–5 compared with bar 2) and IKK-ɛ (Figure 3D; bars 3–5 compared with bar 2), which are kinases which recognize and phosphorylate IRF3 as direct substrate.^48, 49, 50, 51^ Surprisingly, when a constitutively active mutant of IRF3 transcription factor (IRF3 5D), with five inducible phosphorylation sites at Ser/Thr residues mutated to Asp,^52^ was employed, the expression of the M protein no longer quenched the IRF3-induced activation of IFN-β promoter (Figure 3E), suggesting that the inhibitory effect of M protein occurs upstream of IRF3 activation.

**Figure 4:**
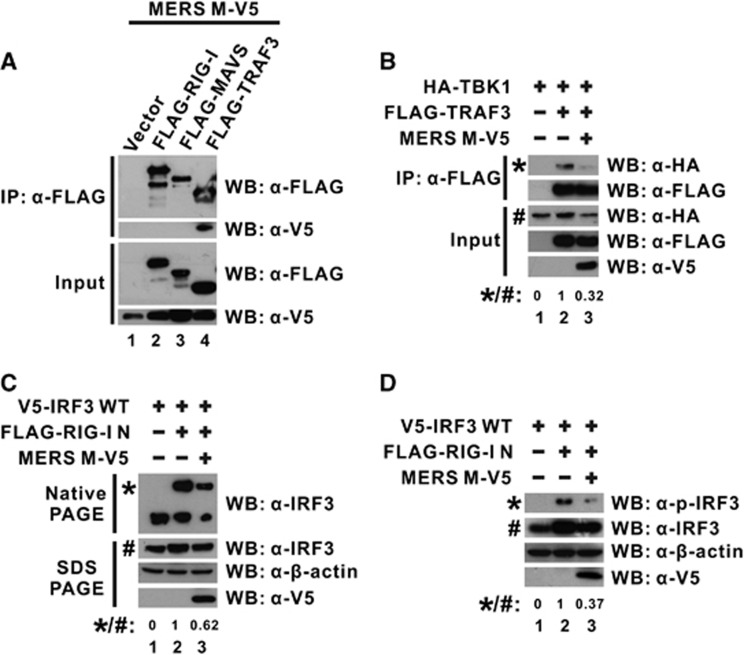
MERS-CoV M protein binds with TRAF3 adapter and perturbs TRAF3–TBK1 interaction. (**A**) HEK-293 cells were transfected with plasmids expressing MERS M-V5 protein and different FLAG-tagged wild-type RLR pathway activators (FLAG-RIG-I, -MAVS and -TRAF3). An empty vector was used as a negative control in lane 1. Cells were collected after 40 h for a co-immunoprecipitation experiment with anti-FLAG antibody. The bound fraction of immunoprecipitates (IP) as well as total cell lysate (as input) were analyzed by western blotting (WB) with anti-FLAG and anti-V5 antibodies. (**B**) HEK-293 cells were transfected with different combinations of expression plasmids for MERS M-V5, FLAG-tagged TRAF3 and HA-tagged TBK1, and collected after 40 h for co-immunoprecipitation experiment with an anti-FLAG antibody to assay for TBK1 recruitment using an anti-HA antibody. (**C**, **D**) HEK-293 cells were transfected with different combinations of expression plasmids for wild-type IRF3, RIG-I N, and MERS M-V5. After 40 h, IRF3 dimerization from cell lysates was visualized by non-denaturing native PAGE followed by western blotting with anti-IRF3 antibodies (**C**). IRF3 phosphorylation was also probed with anti-phopho-IRF3 antibodies (**D**). The expression level of the ectopically expressed proteins was also individually verified in denaturing SDS-PAGE with respective antibodies. The relative band intensity (*/#) of co-immunoprecipitated TBK1 in B, dimeric IRF3 in C or phospho-IRF3 (p-IRF3) in D for each sample was measured using ImageJ software. Retinoic acid-inducible gene-I, RIG-I; RIG-I-like receptor, RLR.

To further analyze the molecular mechanism and consequences through which MERS-CoV M protein exerts its inhibitory effect, we first investigated what signal transducer molecule might interact with the M protein. Several RLR transducers were ectopically expressed with MERS-CoV M protein in cultured cells for a co-immunoprecipitation experiment. When the transducers were precipitated with an anti-FLAG antibody, the M protein was only detected in TRAF3-containing precipitate (Figure 4A; lane 4 compared with lanes 1–3) even though M protein was abundantly expressed in all samples with other transducers (Figure 4A; lower panel for input), indicating the physical association between MERS-CoV M protein and TRAF3.

TRAF3 functions as an adapter that bridges the mitochondrial transducer MAVS with the downstream signaling complex containing TBK1 and IKK-ɛ kinases that are essential for IRF3 activation.^34, 53^ The physical association of the MERS-CoV M protein with TRAF3 (Figure 4A) prompted us to test whether the adapter function of TRAF3 would be particularly affected by M protein. We performed another co-immunoprecipitation experiment to explore the possibility that M protein could perturb the interaction of TRAF3 with the downstream transducer complex. When TRAF3 and TBK1 were ectopically expressed in cultured cells, the detection of TBK1 in TRAF3-immunoprecipitate confirmed the specific recruitment of TBK1 to TRAF3 in the absence of M protein (Figure 4B; lane 2 compared with lane 1). However, when M protein was added to the system, the interaction between TRAF3 and TBK1 was significantly disrupted (Figure 4B; lane 3 compared with lane 2), demonstrating that the physical association of MERS-CoV M protein with TRAF3 perturbs TRAF3–TBK1 interaction.

It was observed that MERS-CoV M protein disrupted TRAF3–TBK1 interaction (Figure 4B), which is required for IRF3 activation. We then evaluated whether IRF3 activation would be affected by the expression of M protein. IRF3 dimerization visualized by non-denaturing native PAGE is a sensitive assay for evaluating direct IRF3 activation.^39^ Therefore, we ectopically expressed IRF3 and M protein with the inducer RIG-I N in cultured cells and subjected cell lysates directly to native PAGE to check for IRF3 dimerization. When the inducer RIG-I N was exclusively co-expressed with IRF3, the detection of an additional slow-migrating band indicated the activation and dimerization of IRF3, which would otherwise be entirely in its monomeric form in the absence of any activator (Figure 4C; lane 2 compared with lane 1). Interestingly, when M protein was expressed, the signal reflecting the dimeric form of IRF3 molecules was significantly diminished, even though the total IRF3 level expressed in all samples was highly comparable as detected by conventional denaturing SDS-PAGE (Figure 4C; lower panel for SDS-PAGE), suggesting that IRF3 activation was greatly inhibited by the MERS-CoV M protein. IRF3 phosphorylation was also suppressed with the expression of MERS M protein in a similar experimental setup (Figure 4D; lane 3 compared with lane 2). Together with other results, MERS-CoV M protein was thought to interact with TRAF3 to perturb TRAF3–TBK1 interaction, which, in turn, affects IRF3 phosphorylation and activation.

### Requirement and sufficiency of the N-terminal transmembrane domains of MERS-CoV M protein for its innate immunosuppressive effect

**Figure 5:**
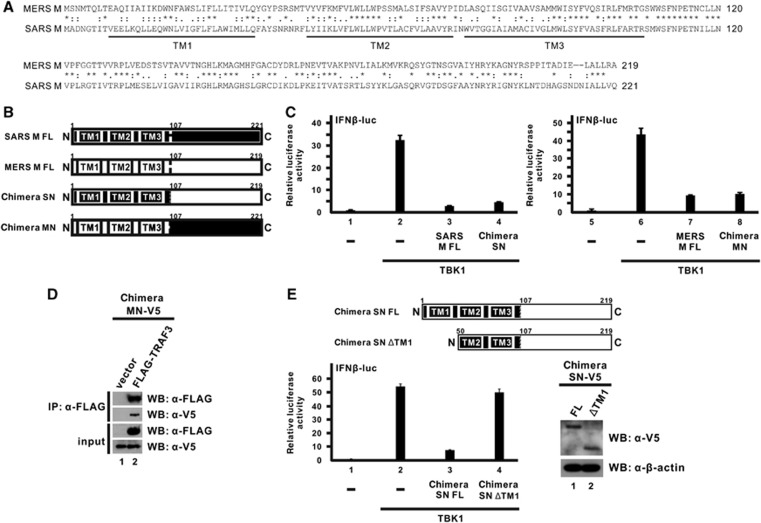
The N-terminal transmembrane domains of the MERS-CoV M protein are sufficient for while its C-terminal domain is devoid of the innate immunosuppressive effect. (**A**) Sequence alignment of MERS-CoV and SARS-CoV M proteins. An asterisk (*) denotes identical residues. A colon (:) and a period (.) represent amino acids with highly and weakly similar physicochemical properties, respectively. The three predicted transmembrane (TM1-3) domains are indicated below the sequences. (**B**) Diagrammatic representation of full-length MERS-CoV and SARS-CoV M proteins (MERS M-FL and SARS M-FL) as well as two chimeras (SN and MN) constructed by swapping the N-terminal and C-terminal domains of the two M proteins with each other. The predicted transmembrane domains and the residue numbers at the breakpoints are indicated. (**C**) HEK-293 cells were co-transfected with plasmids expressing the indicated chimeric protein or the respective full-length M protein together with a TBK1 expression plasmid, a firefly luciferase reporter plasmid driven by IFN-β promoter (IFNβ-luc) and a control *Renilla* luciferase reporter plasmid. Cells were collected after 40 h for dual-luciferase reporter assay. Bars represent the mean of three biological replicates (*n*=3) and error bars indicate their s.d. (**D**) HEK-293 cells were transfected with plasmids expressing V5-MN and FLAG-TRAF3 proteins. An empty vector was used as a negative control in lane 1. Cells were collected after 40 h for a co-immunoprecipitation experiment with an anti-FLAG antibody. The bound fraction of immunoprecipitates (IP) as well as total cell lysate (as input) were analyzed by western blotting (WB) with anti-FLAG and anti-V5 antibodies. (**E**) Diagrammatic representation of chimeric SN full-length (FL) protein and TM1-deletion (ΔTM1) truncation mutant in the upper panel. Inhibition of IFN-β promoter activity by chimeric SN FL or ΔTM1 proteins was measured by luciferase reporter assay in the bottom-left panel as in C. The expression levels of these two proteins were visualized with anti-V5 antibody. β-Actin was also probed as an internal loading control in the bottom-right panel.

Given that the M proteins of both SARS-CoV and MERS-CoV were capable of antagonizing IFN production through highly similar mechanisms,^30^ it will be of interest to analyze the sequence and domain architecture of the two proteins. Sequence alignment of SARS-CoV and MERS-CoV M proteins revealed a strikingly high sequence similarity (>70%) and the presence of three transmembrane domains at the N-termini (Figure 5A). According to the prediction results, we have initially constructed two truncation mutants for MERS-CoV M protein, an N-terminal transmembrane domain-containing mutant and a C-terminal mutant, and tested their inhibitory capacity in suppressing IFN-β expression using a luciferase reporter assay. However, neither exhibited an inhibitory effect (data not shown), possibly due to unstable expression or aberrant localization.

To overcome the inactivity of truncation mutants and to define the inhibitory activity of different domains, we decided to create chimeric proteins using domain swapping between MERS-CoV and SARS-CoV M proteins. Particularly, the SN chimera contains the N-terminal transmembrane domains from SARS-CoV M protein and the C-terminal domain from MERS-CoV M protein, whereas the MN chimera contains the N-terminal transmembrane domains from the MERS-CoV M protein and the C-terminal domain from the SARS-CoV M protein (Figure 5B). The breakpoint was designed to occur immediately after the third predicted transmembrane domain at residue 106 and before the conserved Ser residue in both proteins at residue 107 (Figure 5B).

We next compared the inhibitory effect of these two chimeras and the full-length M proteins on IFN-β expression using the luciferase reporter assay. Our previous study showed that the IFN-antagonizing activity of the SARS-CoV M protein is mediated by its N-terminal transmembrane domains, but the C-terminal domain has no effect.^32^ When we swapped the C-terminal domain of the SARS-CoV M protein with that of the MERS-CoV M protein in the SN chimera, a similar suppressive effect on IFN-β promoter activity was observed (Figure 5C; bar 4 compared with bar 3), consistent with our previous results.^32^ Likewise, when we swapped the C-terminal domain of MERS-CoV M with that of SARS-CoV M protein in MN, the chimera was capable of suppressing IFN-β promoter activity to comparable level (Figure 5C; bar 8 compared with bar 7). Given that the C-terminal domain of SARS-CoV M protein possesses no suppressive effect,^32^ the inhibitory activity of the MN chimera would be predominantly due to the N-terminal domains of MERS-CoV M protein. A biochemical assay also confirmed that the MN chimera maintained the ability to interact with the TRAF3 adapter protein in a co-immunoprecipitation experiment (Figure 5D; lane 2 compared with lane 1). Therefore, we concluded that the N-terminal transmembrane domains of the MERS-CoV M protein are required and sufficient for its innate immunosuppressive activity.

To further determine whether the C-terminal domain of the MERS M protein also possesses IFN-antagonizing activity, we utilized the knowledge that the first transmembrane domain of SARS-CoV M protein is fully responsible for its suppression effect^32^ to construct a truncation mutant lacking the first transmembrane domain in the SN chimera and tested its effect on IFN-β expression using the luciferase reporter assay. This truncation mutant, designated SN ΔTM1, contains only the second and the third transmembrane domains of SARS-CoV M protein at the N terminus fused with the C-terminal domain of MERS-CoV M protein (Figure 5E; upper panel). While the full-length chimeric SN protein was fully competent in suppressing IFN-β promoter activity induced by TBK1, the removal of the first transmembrane domain of SARS-CoV M largely abolished its inhibitory capability (Figure 5E; bottom-left panel, bar 4 compared with bar 3), although both proteins were expressed to a detectable level in cells (Figure 5E; bottom-right panel). It is therefore proposed that the C-terminal domain of MERS-CoV M protein is devoid of and largely dispensable for the IFN-antagonizing activity of MERS-CoV M protein.

## DISCUSSION

In this study, we reported that the MERS-CoV M protein inhibited IRF3 activation, hence IFN expression, by disrupting TRAF3–TBK1 interaction. This innate immunosuppressive activity of the MERS-CoV M protein was due to its conserved N-terminal transmembrane domains. Our mechanistic study complemented the previous work that showed that MERS-CoV M protein had IFN-antagonizing activity.^22^ Both studies demonstrated that MERS-CoV M protein suppressed IRF3 activity but not NF-κB signaling. It is known that the activation of RIG-I and MAVS results in the activation of both IRF3 and NF-κB.^28, 44, 45, 46^ Our results indicated that the MERS-CoV M protein was capable of differentially suppressing the RIG-I-induced activation of IRF3 (Figure 2). This provides further support to the bifurcation of IRF3 and NF-κB signaling subsequent to RIG-I and MAVS activation. Further investigations should elucidate the mechanism by which the MERS-CoV M protein preferentially modulates IRF3 activators such as TBK1, while sparing NF-κB activators such as CARD9.^47^ We provided evidence that the TRAF3–TBK1 interaction as well as IRF3 phosphorylation and dimerization were affected by the MERS-CoV M protein (Figure 4). Our findings fill the knowledge gap by providing novel mechanistic insight into the innate immunosuppressive activity of MERS-CoV M protein.

In our study, TRAF3 was also shown to interact with the MERS-CoV M protein (Figure 4A). In line with this, the adapter function of TRAF3 in TBK1 recruitment and subsequent IRF3 activation was perturbed by MERS-CoV M protein (Figures 4B–D), which plausibly contributed to the IFN antagonism of the MERS-CoV M protein (Figures 1–3). The MERS-CoV M protein is a transmembrane protein that was shown to co-localize with markers of the Golgi apparatus and endoplasmic reticulum (ER)–Golgi intermediate compartments in the perinuclear area.^22^ Although TRAF3 is known to adapt the mitochondrial transducer MAVS, it is not associated with mitochondria but rather with the Golgi apparatus and ER–Golgi intermediate compartments in unstimulated conditions,^30, 54^ rendering it susceptible to interaction with the MERS-CoV M protein. Upon stimulation with ligands or viral infection, TRAF3 appears on membrane-bound fragments originating from Golgi. Retention of TRAF3 in ER-to-Golgi compartments and inability to form Golgi fragments rendered IFN-β expression inefficient.^54^ Therefore, whether TRAF3-containing Golgi fragment formation is affected by MERS-CoV M protein warrants further analysis. This may serve as a novel mechanism by which virus-encoded proteins counteract host IFN production.

MERS-CoV and SARS-CoV are two highly pathogenic coronaviruses that have caused hundreds of deaths. On one hand, the development of relevant prophylactic and therapeutic agents has been well under way.^55, 56, 57^ On the other hand, the identification of the pathogenic factors in these viruses is also in full swing. The M protein is a pathogenic factor by virtue of its IFN-antagonizing property. Both the MERS-CoV and SARS-CoV M proteins were found to suppress IFN production with a highly similar mechanism in which the IRF3-phosphorylating complex of TRAF3·TANK·TBK1/IKK-ɛ was affected by their N-terminal transmembrane domains.^30, 32^ Interestingly, in the case of community-acquired human coronavirus HKU1, which normally causes common cold in infected individuals, its M protein showed no IFN antagonistic property,^32^ further supporting the importance of the M protein in SARS-CoV and MERS-CoV pathogenesis. Using a side-by-side comparison of SARS-CoV and MERS-CoV M proteins, we discovered that the extent by which the MERS-CoV M protein suppressed IFN-β promoter activity was lower than that by SARS-CoV M protein. This difference might be explained by the strengths of the interaction of MERS-CoV M protein with other transducers. Whereas the MERS-CoV M protein was found to be strongly associated with TRAF3, its interaction with TBK1 or IKK-ɛ was undetectable (data not shown). This distinguished MERS-CoV M protein from the SARS-CoV M protein, which interacts potently with every component of the TRAF3·TANK·TBK1/IKK-ɛ complex.^30^ Further investigations are required to shed light on how the interaction of the M protein with TRAF3 complex might influence MERS-CoV pathogenesis.

Coronaviruses encode multiple proteins to counteract the host innate antiviral response.^58, 59, 60^ MERS-CoV is no exception. Several MERS-CoV-encoded proteins have been identified to be IFN antagonists. We, and others, have characterized at least three IFN-antagonizing proteins encoded by MERS-CoV. In addition to the M protein reported in this study, ORF4a is a dsRNA-binding protein, which directly inhibits RLR activation induced by dsRNA and/or the protein co-activator PACT.^24, 25^ In addition, our unpublished data also revealed that ORF4b is a potent IFN antagonist. This is in line with findings by other groups although the mechanistic details of its action have not well documented.^22, 23^ One recent report suggested that ORF4b might not only interact directly with TBK1/IKKɛ in the cytoplasm but also perturb IFN production in the nucleus through an as yet unknown mechanism.^61^ Nevertheless, how M, ORF4a, ORF4b and the other IFN-antagonizing proteins of MERS-CoV coordinate with each other to modulate the host innate antiviral response to facilitate viral replication and propagation remains elusive.

## References

[bib1] Zaki AM, van Boheemen S, Bestebroer TM, Osterhaus AD, Fouchier RA. Isolation of a novel coronavirus from a man with pneumonia in Saudi Arabia. N Engl J Med 2012; 367: 1814–1820. 23075143 10.1056/NEJMoa1211721

[bib2] Centers for Disease Control and Prevention (CDC). Severe respiratory illness associated with a novel coronavirus-Saudi Arabia and Qatar, 2012. MMWR Morb Mortal Wkly Rep 2012; 61: 820. 23051613

[bib3] Bermingham A, Chand MA, Brown CS et al. Severe respiratory illness caused by a novel coronavirus, in a patient transferred to the United Kingdom from the Middle East, September 2012. Euro Surveill 2012; 17: 20290. 23078800

[bib4] Mailles A, Blanckaert K, Chaud P et al. First cases of Middle East Respiratory Syndrome Coronavirus (MERS-CoV) infections in France, investigations and implications for the prevention of human-to-human transmission, France, May 2013. Euro Surveill 2013; 18: 20502. 23787161

[bib5] Puzelli S, Azzi A, Santini MG et al. Investigation of an imported case of Middle East Respiratory Syndrome Coronavirus (MERS-CoV) infection in Florence, Italy, May to June 2013. Euro Surveill 2013; 18: 20564. 23987829 10.2807/1560-7917.es2013.18.34.20564

[bib6] Tsiodras S, Baka A, Mentis A et al. A case of imported Middle East Respiratory Syndrome coronavirus infection and public health response, Greece, April 2014. Euro Surveill 2014; 19: 20782. 24786258 10.2807/1560-7917.es2014.19.16.20782

[bib7] Bialek SR, Allen D, Alvarado-Ramy F et al. First confirmed cases of Middle East respiratory syndrome coronavirus (MERS-CoV) infection in the United States, updated information on the epidemiology of MERS-CoV infection, and guidance for the public, clinicians, and public health authorities - May 2014. MMWR 2014; 63: 431–436. 24827411 PMC5779407

[bib8] Chan JF, Lau SK, To KK, Cheng VC, Woo PC, Yuen KY. Middle East respiratory syndrome coronavirus: another zoonotic betacoronavirus causing SARS-like disease. Clin Microbiol Rev 2015; 28: 465–522. 25810418 10.1128/CMR.00102-14 PMC4402954

[bib9] Hui DS, Perlman S, Zumla A. Spread of MERS to South Korea and China. Lancet Respir Med 2015; 3: 509–510. 26050550 10.1016/S2213-2600(15)00238-6 PMC7128695

[bib10] World Health Organization (WHO). Middle East respiratory syndrome coronavirus (MERS-CoV). 2015. WHO: Geneva, 2015. Available at http://www.who.int/emergencies/mers-cov/en accessed 5 November 2015.

[bib11] de Groot RJ, Baker SC, Baric RS et al. Middle East respiratory syndrome coronavirus (MERS-CoV): announcement of the Coronavirus Study Group. J Virol 2013; 87: 7790–7792. 23678167 10.1128/JVI.01244-13 PMC3700179

[bib12] Lu L, Liu Q, Du L, Jiang S. Middle East respiratory syndrome coronavirus (MERS-CoV): challenges in identifying its source and controlling its spread. Microbes Infect 2013; 15: 625–629. 23791956 10.1016/j.micinf.2013.06.003 PMC7110483

[bib13] van Boheemen S, de Graaf M, Lauber C et al. Genomic characterization of a newly discovered coronavirus associated with acute respiratory distress syndrome in humans. MBio 2012; 3: e00473–e00512. 23170002 10.1128/mBio.00473-12 PMC3509437

[bib14] Durai P, Batool M, Shah M, Choi S. Middle East respiratory syndrome coronavirus: transmission, virology and therapeutic targeting to aid in outbreak control. Exp Mol Med 2015; 47: e181. 26315600 10.1038/emm.2015.76 PMC4558490

[bib15] Zielecki F, Weber M, Eickmann M et al. Human cell tropism and innate immune system interactions of human respiratory coronavirus EMC compared to those of severe acute respiratory syndrome coronavirus. J Virol 2013; 87: 5300–5304. 23449793 10.1128/JVI.03496-12 PMC3624328

[bib16] Chan RW, Chan MC, Agnihothram S et al. Tropism of and innate immune responses to the novel human betacoronavirus lineage C virus in human *ex vivo* respiratory organ cultures. J Virol 2013; 87: 6604–6614. 23552422 10.1128/JVI.00009-13 PMC3676115

[bib17] Lau SK, Lau CC, Chan KH et al. Delayed induction of proinflammatory cytokines and suppression of innate antiviral response by the novel Middle East respiratory syndrome coronavirus: implications for pathogenesis and treatment. J Gen Virol 2013; 94: 2679–2690. 24077366 10.1099/vir.0.055533-0

[bib18] Zhou J, Chu H, Li C et al. Active replication of Middle East respiratory syndrome coronavirus and aberrant induction of inflammatory cytokines and chemokines in human macrophages: implications for pathogenesis. J Infect Dis 2014; 209: 1331–1342. 24065148 10.1093/infdis/jit504 PMC7107356

[bib19] Faure E, Poissy J, Goffard A et al. Distinct immune response in two MERS-CoV-infected patients: can we go from bench to bedside? PLoS One 2014; 9: e88716. 24551142 10.1371/journal.pone.0088716 PMC3925152

[bib20] Chan JF, Yao Y, Yeung ML et al. Treatment With Lopinavir/Ritonavir or interferon-β1b improves outcome of MERS-CoV infection in a nonhuman primate model of common marmoset. J Infect Dis 2015; 212: 1904–1913. 26198719 10.1093/infdis/jiv392 PMC7107395

[bib21] Agrawal AS, Garron T, Tao X et al. Generation of a transgenic mouse model of Middle East respiratory syndrome coronavirus infection and disease. J Virol 2015; 89: 3659–3670. 25589660 10.1128/JVI.03427-14 PMC4403411

[bib22] Yang Y, Zhang L, Geng H et al. The structural and accessory proteins M, ORF 4a, ORF 4b, and ORF 5 of Middle East respiratory syndrome coronavirus (MERS-CoV) are potent interferon antagonists. Protein Cell 2013; 4: 951–961. 24318862 10.1007/s13238-013-3096-8 PMC4875403

[bib23] Matthews KL, Coleman CM, van der Meer Y, Snijder EJ, Frieman MB. The ORF4b-encoded accessory proteins of Middle East respiratory syndrome coronavirus and two related bat coronaviruses localize to the nucleus and inhibit innate immune signalling. J Gen Virol 2014; 95: 874–882. 24443473 10.1099/vir.0.062059-0 PMC3973478

[bib24] Niemeyer D, Zillinger T, Muth D et al. Middle East respiratory syndrome coronavirus accessory protein 4a is a type I interferon antagonist. J Virol 2013; 87: 12489–12495. 24027320 10.1128/JVI.01845-13 PMC3807936

[bib25] Siu KL, Yeung ML, Kok KH et al. Middle east respiratory syndrome coronavirus 4a protein is a double-stranded RNA-binding protein that suppresses PACT-induced activation of RIG-I and MDA5 in the innate antiviral response. J Virol 2014; 88: 4866–4876. 24522921 10.1128/JVI.03649-13 PMC3993821

[bib26] Yang X, Chen X, Bian G et al. Proteolytic processing, deubiquitinase and interferon antagonist activities of Middle East respiratory syndrome coronavirus papain-like protease. J Gen Virol 2014; 95: 614–626. 24362959 10.1099/vir.0.059014-0

[bib27] Chu H, Zhou J, Wong BH et al. Productive replication of Middle East respiratory syndrome coronavirus in monocyte-derived dendritic cells modulates innate immune response. Virology 2014; 454-455: 197–205. 24725946 10.1016/j.virol.2014.02.018 PMC7111975

[bib28] Yoneyama M, Kikuchi M, Natsukawa T et al. The RNA helicase RIG-I has an essential function in double-stranded RNA-induced innate antiviral responses. Nat Immunol 2004; 5: 730–737. 15208624 10.1038/ni1087

[bib29] Kato H, Sato S, Yoneyama M et al. Cell type-specific involvement of RIG-I in antiviral response. Immunity 2005; 23: 19–28. 16039576 10.1016/j.immuni.2005.04.010

[bib30] Siu KL, Kok KH, Ng MH et al. Severe acute respiratory syndrome coronavirus M protein inhibits type I interferon production by impeding the formation of TRAF3·TANK·TBK1/IKKɛ complex. J Biol Chem 2009; 284: 16202–16209. 19380580 10.1074/jbc.M109.008227 PMC2713514

[bib31] Neuman BW, Kiss G, Kunding AH et al. A structural analysis of M protein in coronavirus assembly and morphology. J Struct Biol 2011; 174: 11–22. 21130884 10.1016/j.jsb.2010.11.021 PMC4486061

[bib32] Siu KL, Chan CP, Kok KH, Woo PCY, Jin DY. Suppression of innate antiviral response by severe acute respiratory syndrome coronavirus M protein is mediated through the first transmembrane domain. Cell Mol Immunol 2014; 11: 141–149. 24509444 10.1038/cmi.2013.61 PMC4003381

[bib33] Doyle S, Vaidya S, O'Connell R et al. IRF3 mediates a TLR3/TLR4-specific antiviral gene program. Immunity 2002; 17: 251–263. 12354379 10.1016/s1074-7613(02)00390-4

[bib34] Guo B, Cheng G. Modulation of the interferon antiviral response by the TBK1/IKKi adapter protein TANK. J Biol Chem 2007; 282: 11817–11826. 17327220 10.1074/jbc.M700017200

[bib35] Ng MH, Ho TH, Kok KH, Siu KL, Li J, Jin DY. . MIP-T3 is a negative regulator of innate type I IFN response. J Immunol 2011; 187: 6473–6482. 22079989 10.4049/jimmunol.1100719

[bib36] Kok KH, Lui PY, Ng MH, Siu KL, Au SW, Jin DY. The double-stranded RNA-binding protein PACT functions as a cellular activator of RIG-I to facilitate innate antiviral response. Cell Host Microbe 2011; 9: 299–309. 21501829 10.1016/j.chom.2011.03.007

[bib37] Chaudhary V, Zhang S, Yuen KS et al. Suppression of type I and type III interferon signalling by NSs protein of severe fever-with-thrombocytopenia syndrome virus through inhibition of STAT1 phosphorylation and activation. J Gen Virol 2015; 96: 3204–3211. 26353965 10.1099/jgv.0.000280

[bib38] Siu KL, Chan CP, Kok KH, Woo PC, Jin DY. Comparative analysis of the activation of unfolded protein response by spike proteins of severe acute respiratory syndrome coronavirus and human coronavirus HKU1. Cell Biosci 2014; 4: 3. 24410900 10.1186/2045-3701-4-3 PMC3930072

[bib39] Iwamura T, Yoneyama M, Yamaguchi K et al. Induction of IRF-3/-7 kinase and NF-κB in response to double-stranded RNA and virus infection: common and unique pathways. Genes Cells 2001; 6: 375–388. 11318879 10.1046/j.1365-2443.2001.00426.x

[bib40] Kew C, Lui PY, Chan CP et al. Suppression of PACT-induced type I interferon production by herpes simplex virus 1 Us11 protein. J Virol 2013; 87: 13141–13149. 24067967 10.1128/JVI.02564-13 PMC3838286

[bib41] Sievers F, Wilm A, Dineen D et al. Fast, scalable generation of high-quality protein multiple sequence alignments using Clustal Omega. Mol Syst Biol 2011; 7: 539. 21988835 10.1038/msb.2011.75 PMC3261699

[bib42] Deber CM, Wang C, Liu LP et al. TM Finder: a prediction program for transmembrane protein segments using a combination of hydrophobicity and nonpolar phase helicity scales. Protein Sci 2001; 10: 212–219. 11266608 10.1110/ps.30301 PMC2249854

[bib43] Thanos D, Maniatis T. Virus induction of human IFNβ gene expression requires the assembly of an enhanceosome. Cell 1995; 83: 1091–1100. 8548797 10.1016/0092-8674(95)90136-1

[bib44] Seth RB, Sun L, Ea CK, Chen ZJ. Identification and characterization of MAVS, a mitochondrial antiviral signaling protein that activates NF-κB and IRF 3. Cell 2005; 122: 669–682. 16125763 10.1016/j.cell.2005.08.012

[bib45] Kawai T, Takahashi K, Sato S et al. IPS-1, an adapter triggering RIG-I- and Mda5-mediated type I interferon induction. Nat Immunol 2005; 6: 981–988. 16127453 10.1038/ni1243

[bib46] Xu LG, Wang YY, Han KJ, Li LY, Zhai Z et al. VISA is an adapter protein required for virus-triggered IFN-β signaling. Mol Cell 2005; 19: 727–740. 16153868 10.1016/j.molcel.2005.08.014

[bib47] Poeck H, Bscheider M, Gross O et al. Recognition of RNA virus by RIG-I results in activation of CARD9 and inflammasome signaling for interleukin 1β production. Nat Immunol 2010; 11: 63–69. 19915568 10.1038/ni.1824

[bib48] Fitzgerald KA, McWhirter SM, Faia KL et al. IKKɛ and TBK1 are essential components of the IRF3 signaling pathway. Nat Immunol 2003; 4: 491–496. 12692549 10.1038/ni921

[bib49] McWhirter SM, Fitzgerald KA, Rosains J, Rowe DC, Golenbock DT, Maniatis T. IFN-regulatory factor 3-dependent gene expression is defective in Tbk1-deficient mouse embryonic fibroblasts. Proc Natl Acad Sci USA 2004; 101: 233–238. 14679297 10.1073/pnas.2237236100 PMC314168

[bib50] Sankar S, Chan H, Romanow WJ, Li J, Bates RJ. IKK-i signals through IRF3 and NFkappaB to mediate the production of inflammatory cytokines. Cell Signal 2006; 18: 982–993. 16199137 10.1016/j.cellsig.2005.08.006

[bib51] Liu S, Cai X, Wu J et al. Phosphorylation of innate immune adapter proteins MAVS, STING, and TRIF induces IRF3 activation. Science 2015; 347: aaa2630. 25636800 10.1126/science.aaa2630

[bib52] Lin R, Heylbroeck C, Pitha PM, Hiscott J. Virus-dependent phosphorylation of the IRF-3 transcription factor regulates nuclear translocation, transactivation potential, and proteasome-mediated degradation. Mol Cell Biol 1998; 18: 2986–2996. 9566918 10.1128/mcb.18.5.2986 PMC110678

[bib53] Saha SK, Pietras EM, He JQ et al. Regulation of antiviral responses by a direct and specific interaction between TRAF3 and Cardif. EMBO J 2006; 25: 3257–3263. 16858409 10.1038/sj.emboj.7601220 PMC1523175

[bib54] van Zuylen WJ, Doyon P, Clément JF et al. Proteomic profiling of the TRAF3 interactome network reveals a new role for the ER-to-Golgi transport compartments in innate immunity. PLoS Pathog 2012; 8: e1002747. 22792062 10.1371/journal.ppat.1002747 PMC3390413

[bib55] Chen WH, Du L, Chag SM et al. Yeast-expressed recombinant protein of the receptor-binding domain in SARS-CoV spike protein with deglycosylated forms as a SARS vaccine candidate. Hum Vaccin Immunother 2014; 10: 648–658. 24355931 10.4161/hv.27464 PMC4130269

[bib56] Lu L, Liu Q, Zhu Y et al. Structure-based discovery of Middle East respiratory syndrome coronavirus fusion inhibitor. Nat Commun 2014; 5: 3067. 24473083 10.1038/ncomms4067 PMC7091805

[bib57] Ying T, Li H, Lu L, Dimitrov DS, Jiang S. Development of human neutralizing monoclonal antibodies for prevention and therapy of MERS-CoV infections. Microbes Infect 2015; 17: 142–148. 25456101 10.1016/j.micinf.2014.11.008 PMC4308519

[bib58] Totura AL, Baric RS. SARS coronavirus pathogenesis: host innate immune responses and viral antagonism of interferon. Curr Opin Virol 2012; 2: 264–275. 22572391 10.1016/j.coviro.2012.04.004 PMC7102726

[bib59] Kindler E, Thiel V. To sense or not to sense viral RNA—essentials of coronavirus innate immune evasion. Curr Opin Microbiol 2014; 20: 69–75. 24908561 10.1016/j.mib.2014.05.005 PMC7108419

[bib60] Wong LYR, Lui PY, Jin DY. A molecular arms race between host innate antiviral response and emerging human coronaviruses. Virol Sin 2016; 31: 12–23. 26786772 10.1007/s12250-015-3683-3 PMC7090626

[bib61] Yang Y, Ye F, Zhu N et al. Middle East respiratory syndrome coronavirus ORF4b protein inhibits type I interferon production through both cytoplasmic and nuclear targets. Sci Rep 2015; 5: 17554. 26631542 10.1038/srep17554 PMC4668369

